# Proposal for delta check limits of frequently requested hormones using real-world data

**DOI:** 10.11613/BM.2025.010704

**Published:** 2025-02-15

**Authors:** Sunghwan Shin, Shinae Yu, Sollip Kim, Soo Jin Yoo, Eun-Jung Cho, Jae-Woo Chung

**Affiliations:** 1 Department of Laboratory Medicine, Ilsan Paik Hospital, Inje University College of Medicine, Goyang, Republic of Korea; 2 Department of Laboratory Medicine, Haeundae Paik Hospital, Inje University College of Medicine, Busan, Republic of Korea; 3 Department of Laboratory Medicine, Asan Medical Center, University of Ulsan College of Medicine, Seoul, Republic of Korea; 4 Department of Laboratory Medicine, Sanggye Paik Hospital, Inje University College of Medicine, Seoul, Republic of Korea; 5 Department of Laboratory Medicine, Hallym University Dongtan Sacred Heart Hospital, Hallym University College of Medicine, Hwaseong, Republic of Korea; 6 Departments of Laboratory Medicine, Dongguk University Ilsan Hospital, Goyang, Republic of Korea

**Keywords:** delta check, hormone tests, laboratory tests, result verification, postanalytical phase management

## Abstract

**Introduction:**

Research on delta check limits (DCLs) for hormones is limited, yet some laboratories apply arbitrary DCLs. We aimed to propose DCLs for commonly requested hormones.

**Materials and methods:**

This study analyzed 59,657 paired results for adrenocorticotropic hormone (ACTH), cortisol, parathyroid hormone (PTH), prolactin, insulin, testosterone, and thyroglobulin from five Korean university hospitals. Delta check limits were established using the absolute delta difference (absDD) and absolute delta percent change (absDPC) with 5% cutoff for inpatients/emergencies (IE), outpatients (O) and both (combined; mean of them). Proportions outside the DCLs were compared across groups.

**Results:**

Using absDD and absDPC, each group’s DCLs showed 4.3% to 6.4% of values outside the DCLs, aligning with the 5% cutoff (excluding group IE for insulin, testosterone, and thyroglobulin due to < 1000 data pairs). Delta check limits of absDD differed between groups for ACTH, cortisol, PTH, and prolactin, while for absDPC, differences were seen only for ACTH and prolactin. Cross-validation revealed IE and O groups differed outside DCLs of absDD for ACTH, cortisol, and PTH, but only ACTH with absDPC. Combined DCLs of absDD showed ACTH and cortisol exceeded limits in 7.2% and 9.0% in IE, but only 2.6% and 0.6% in O. With absDPC, ACTH differed (10.4% in IE, 2.8% in O), while cortisol, PTH, and prolactin ranged from 4.0% to 6.1%.

**Conclusions:**

Combined DCLs of absDPC are recommended for cortisol, PTH, and prolactin, while ACTH requires separate DCLs on clinical settings. These DCLs from real-world data provide a foundation for establishing DCLs of hormones in clinical laboratories.

## Graphical abstract

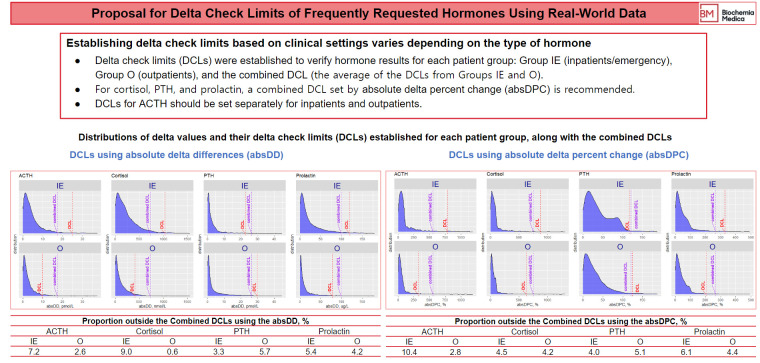

## Highlights

• Delta check limits were established for frequently requested hormones

• An absolute delta percent check is recommended to verify common hormones

• Delta check limits for adrenocorticotropic hormone should be set separately for inpatients and outpatients

• This foundational data helps set delta check limits for hormones in laboratories

## Introduction

Hormones play significant roles in regulating various physiological processes within the body. They are intricately linked to the body’s various physiological states, and changes in their concentrations can provide critical insights into specific disease conditions. In clinical practice, the concentrations of particular hormones serve as crucial indicators for assessing a patient’s health status and response to treatment (*1*, *2*). Even the smallest changes in hormone concentrations may be actively considered by clinicians, leading to treatment plan adjustments. For example, after a thyroidectomy for thyroid cancer, thyroglobulin, a precursor to thyroid hormones, is regularly monitored to ensure it remains below the limit of detection. The rise in thyroglobulin concentration could be indicative of a recurrence of thyroid cancer, requiring further workup (*3*). Parathyroid hormone (PTH) is a surrogate marker in the clinical prediction of renal osteodystrophy and fracture in chronic kidney disease. Regular monitoring of PTH is crucial for therapeutic decision-making, with an increase in PTH leading to medical treatment and possibly parathyroidectomy as the last option (*4*).

Accurate and error-free reporting of hormonal test results is vital. However, the laboratory results can be compromised by various causes including sample misidentifications, mechanical errors, or human mistakes, thereby potentially leading to incorrect diagnoses or treatments. Many laboratories try to detect errors by monitoring patient test results and by performing various quality management activities to ensure that the reported results are accurate (*5*-*9*). One of the most commonly used methods for verifying patients’ results in postanalytical phases is the delta check (*10*, *11*). The delta check method compares a patient’s current test results with previous results to detect significant changes that may indicate errors (*12*). However, due to the inherent fluctuations in hormone concentrations caused by various physiological or pathological factors, determining the applicability of delta checks for hormone tests is challenging. This likely explains why delta check limits (DCLs) for hormone tests are rarely published. However, some laboratory practitioners routinely perform delta checks using arbitrary cutoffs, resulting in unnecessary checks that increase the laboratory’s workload (*11*, *13*, *14*).

We previously evaluated and validated DCLs for thyroid hormones in a previous multicenter study, recommending different DCLs for health checkup recipients compared with other patient groups (inpatients, outpatients, *etc*.) (*15*). In the current study, we aimed to establish DCLs, defined as limits that can be feasibly applied in routine clinical laboratory settings without the need for additional clinical information, for frequently requested hormones, excluding thyroid hormones. We collected multicenter data to determine DCLs based on the result distributions for tests including adrenocorticotropic hormone (ACTH), cortisol, insulin, PTH, prolactin, testosterone, and thyroglobulin. Our objective was to investigate whether separate DCLs should be applied for distinct clinical situations, such as inpatients and outpatients, or if a combined DCL could effectively address both settings.

## Materials and methods

### Materials

This study was conducted across five clinical laboratories of university-affiliated hospitals in the Republic of Korea. All five laboratories were accredited by the Laboratory Accreditation Program of the Laboratory Medicine Foundation and participated in external quality assessment (EQA) programs of the Korean Association of External Quality Assessment Service (KEQAS) in Korea and have received acceptable scores (*16*, *17*). All paired results (current and previous) for ACTH, cortisol, insulin, PTH, prolactin, testosterone and thyroglobulin were retrospectively collected. The current results were tested between January 2020 and August 2022 (a span of 32 months). The Institutional Review Board of each institution approved this study (2022-11-030, HDT 2022-11-009, HPIRB 2022-09-017, ISPAIK 2022-09-031, SGPAIK 2023-01-013), and the investigation was performed in compliance with the principles of the Helsinki Declaration and its amendments. Informed consent was waived due to the retrospective study design and anonymized data.

### Methods

Each test was performed on a Roche Cobas C-8000 instrument (Roche Diagnostics, GmbH, Mannheim, Germany) with dedicated calibrators and reagents (Elecsys ACTH, Elecsys Cortisol II, Elecsys insulin, Elecsys PTH, Elecsys Prolactin II, Elecsys Testosterone II and Elecsys Thyroglobulin II) at each institution. The ACTH samples were collected in K2-EDTA tubes (Becton Dickinson, Plymouth, UK). For thyroglobulin, testosterone, prolactin, insulin, cortisol, and PTH, samples were collected in VACUETTE CAT Serum Separator Clot Activators (Greiner Bio-One GmbH, Kremsmünster, Austria) or BD Vacutainer SST II Advance (Becton Dickinson, Franklin Lakes, USA). All samples were immediately centrifuged at 1650-1800xg for 10 minutes to separate plasma and serum. Tests were conducted immediately within 2 hours, if immediate testing was impossible, samples were refrigerated at + 4 °C and tested within a maximum of 72 hours. The analytical measurement intervals were 0.330-440 pmol/L (1.5-2000 pg/mL) for ACTH, 1.5-1750 nmol/L (0.054-63.4 μg/dL) for cortisol, 0.127-530 pmol/L (1.2-5000 pg/mL) for PTH, 0.094-470 ug/L (2-10,000 µlU/mL) for prolactin, 2.78-6945 pmol/L (0.4-1000 μIU/mL) for insulin, 0.087-52.0 nmol/L (2.50-1500 ng/dL) for testosterone and 0.04-500 ug/L (0.04-500 ng/mL) for thyroglobulin. Results with inequality signs, non-numeric values, and time differences between the current and previous tests of < 24 h or > 3 years were excluded. When multiple paired results were obtained from the same patient, each set was treated as an independent observation and included in the analysis without exclusion.

**Figure 1:**
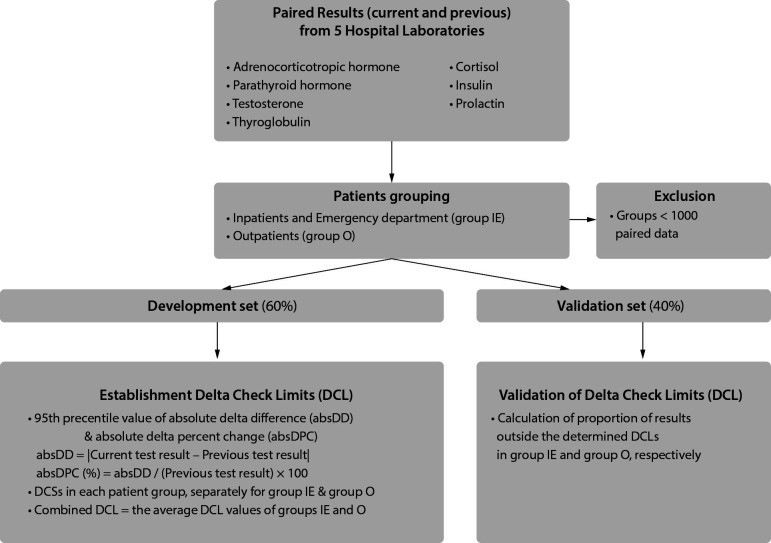
Flowchart of data processing for the establishment and validation of delta check limits for hormones

The analysis was limited to adults aged ≥ 19 years. Information was limited to the patients’ status as inpatients, outpatients, or emergency department cases, along with the requesting department. Thus, detailed clinical situations or underlying conditions of the patients were not available. Patients were categorized into two groups for analysis: one comprising inpatients and patients in the emergency department (group IE), and the other comprising outpatients (group O) (Figure 1). Among the 14 datasets analyzed (two groups for each of the seven tests), those with < 1000 current-previous paired results were excluded from the analysis, as a smaller sample size reduces statistical robustness. The total data was randomly divided into a development set (D set) and a validation set (V set) at a 6:4 ratio within each IE and O group, balancing the need for robust DCL determination in the D set with sufficient data for validation in the V set. The D set was used to determine the DCLs, which include the absolute delta difference (absDD) and absolute delta percent change (absDPC), calculated using the paired data for each test. The variable absDD was calculated by obtaining the absolute difference between the current and previous test results and was expressed in the units of measurement for each hormone. The absDPC was computed by dividing the absDD by the previous test result and expressed as a percentage. The formulas for absDD and absDPC were as follows: absDD = current test result - previous test result, absDPC (%) = absDD / (previous test result) × 100.

The upper 95th percentile of the absDD and absDPC distribution was set as the DCL for each test in both groups IE and O. Quantile regression was performed between groups IE and O to determine if there was a significant difference between the DCLs. Additionally, combined DCLs were established based on the average values from groups IE and O to evaluate the need for differentiation between groups IE and O. To eliminate the effect of varying sample sizes in groups IE and O, we used arithmetic means instead of weighted averages. Additionally, the 2.5th and 97.5th percentiles for both DD and DPC, stratified by male and female.

The V set was used to validate the determined DCLs. The proportion of results outside the determined DCLs was calculated within each group. The DCLs were cross-validated by applying the determined DCLs of group IE to group O, and *vice versa*. The purpose of this cross-validation was to assess whether the determined DCLs established for groups IE and O were interchangeable. The expected proportion outside the established DCL is set at 5%, as we have defined the DCL using the 95th percentile.

To validate the combined DCLs, groups IE and O were validated using the combined DCLs. A comparison of the validation results of groups IE and O using combined DCLs was performed using Chi-squared analysis.

The reference change value (RCV) was calculated using the asymmetrical RCV formula, determining separate RCVs for increases and decreases, with the aid of the RCV calculator provided by the European Federation of Clinical Chemistry and Laboratory Medicine (EFLM) biological variation database (*18*, *19*). Afterward, two-sided approaches Z-scores (1.96 for 95%, and 2.57 for 99%) were used. The analytical imprecision (CV_A_) used in the calculations was determined as the average of CV_A_s obtained from quality control (QC) materials at each hospital. The within-subject biological variation (CV_I_) was obtained from the EFLM biological variation database (*19*). The RCV for ACTH could not be calculated due to the absence of a CV_I_ value provided by the EFLM database.

### Statistical analysis

All statistical analyses were performed using R (Project for Statistical Computing, version 4.2.2, available from: http://cran.r-project.org). Quantile regression, using the “quantreg” package, and Chi-squared analysis, using the “gtsummary” package, were conducted in R. P < 0.05 was considered statistically significant (*20*, *21*).

## Results

A total of 59,657 paired test results for ACTH, cortisol, insulin, PTH, prolactin, testosterone, and thyroglobulin were gathered. The percentage of samples received between 6 a.m. and 12 p.m. for each test were as follows: ACTH (75.6%), cortisol (78.2%), PTH (71.3%), prolactin (74.7%), insulin (95.3%), testosterone (88.6%), and thyroglobulin (80.1%). Table 1 provides the general characteristics of the collected data.

**Table 1:** General characteristics of the data

	**ACTH** **(pmol/L)**			**Cortisol** **(nmol/L)**			**PTH** **(pmol/L)**			**Prolactin** **(μg/L)**				**Insulin** **(pmol/L)**		**Testosterone (nmol/L)**	**Thyroglobulin (μg/L)**
	**IE**	**O**	P *	**IE**	**O**	P *	**IE**	**O**	P *	**IE**	**O**	P *		**O**		**O**	**O**
Total number of paired results (N)	1361	2800		2305	4855		1466	19,804		1983	3874			5515		4180	11,514
Sex, female (N,%)	735 (54)	1769 (63)	< 0.001	1210 (53)	2938 (61)	< 0.001	971 (66)	12,511 63)	0.019	1301 (66)	3060 (79)	< 0.001		2135 (39)		259 (6.2)	8586 (75)
Age, median (min-max)	73 (19-99)	62 (19-98)	< 0.001	71 (19-99)	63 (19-98)	< 0.001	55 (21-96)	60 (19-99)	< 0.001	38 (19-98)	42 (19-89)	< 0.001		60 (19-93)		68 (19-98)	53 (19-98)
Test result, median (IQR)	4.8 (2.2-8.9)	4.6 (2.7-7.7)	0.381	325.7 (173.1-488.5)	187.7 (58.0-298.1)	< 0.001	3.2 (1.4-5.8)	6.1 (2.9-23.3)	< 0.001	38.8 (15.9-84.9)	17.1 (9.4-33.1)	< 0.001		57.0 (35.4-92.4)		0.1 (0.0-0.5)	2.6 (0.5-7.3)
Institutions, N (%)			< 0.001			< 0.001			< 0.001			< 0.001					
A	207 (15.2)	626 (22.4)		278 (12.1)	877 (18.1)		71 (4.8)	2835 (14.3)		108 (5.4)	553 (14.3)				3,074 (55.7)	236 (5.6)	762 (6.6)
B	62 (4.6)	252 (9.0)		185 (8.0)	708 (14.6)		207 (14.1)	3826 (19.3)		955 (48.2)	1036 (26.7)				1800 (32.6)	1164 (27.8)	1930 (16.8)
C	1092 (80.2)	1922 (68.6)		1584 (68.7)	2611 (53.8)		874 (59.6)	9968 (50.3)		353 (17.8)	1864 (48.1)				499 (9.0)	2780 (66.5)	5,459 (47.4)
D	0 (0.0)	0 (0.0)		258 (11.2)	659 (13.6)		314 (21.4)	3175 (16.0)		567 (28.6)	421 (10.9)				142 (2.6)	0 (0.0)	712 (6.2)
E	0 (0.0)	0 (0.0)		0 (0.0)	0 (0.0)		0 (0.0)	0 (0.0)		0 (0.0)	0 (0.0)				0 (0.0)	0 (0.0)	2651 (23.0)
Delta interval in days, median (IQR)	34 (10-173)	147 (88-209)	< 0.001	27 (8-130)	120 (77-198)	< 0.001	15 (4-28)	84 (28-180)	< 0.001	12 (7-49)	168 (90-222)	< 0.001		179 (113-345)		167 (91-233)	174 (94-189)
*Statistical significance was determined using Pearson’s Chi-squared test or Wilcoxon rank sum test between inpatients/emergency and outpatients. ACTH - adrenocorticotropic hormone. PTH - parathyroid hormone. IQR - interquartile range. IE - inpatients/emergency. O - outpatients. P < 0.05 was considered statistically significant.																	

**Figure 2:**
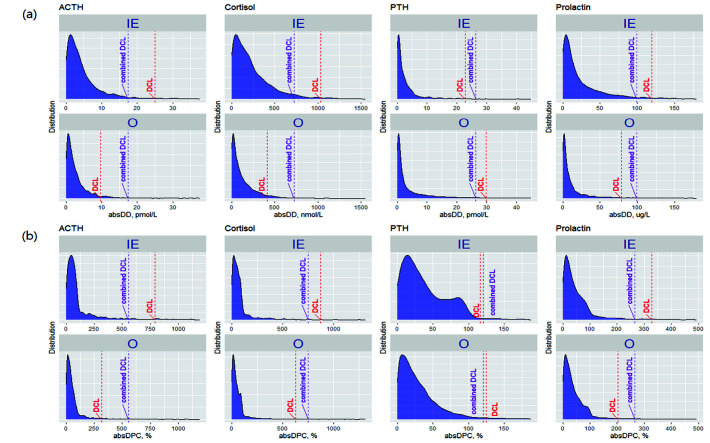
Distributions of absolute delta difference (absDD) (A) and absolute delta percent change (absDPC) (B) in inpatients/emergency (group IE) and outpatients (group O) for ACTH, cortisol, PTH and prolactin. Red dashed lines indicate the delta check limits (DCLs) determined for each group, while purple dashed lines indicate combined DCLs calculated as their averages. ACTH - adrenocorticotropic hormone. PTH - parathyroid hormone.

Figure 2 shows the distribution of absDD and absDPC values for ACTH, cortisol, PTH and prolactin in group IE and group O, along with the DCLs determined for each group and the combined DCLs calculated as their average. Table 2 presents these DCL values and the proportions outside the DCLs in each group of validation sets when applying the group-specific DCLs, those of the other groups, and the combined DCLs.

**Table 2:** Separate and combined DCLs with validation of the DCLs between groups using absDD and absDPC

		**DCLs using the absDD**	**DCLs using absDPC**

**Parameter, unit**	**Group**	**DCLs**	P*	**Proportion outside DCLs in IE group, %**	**Proportion outside DCLs, in O group, %**	**P^†^**	**DCLs, %**	P*	**Proportion outside DCLs in IE group %**	**Proportion outside DCLs, in O group %**	**P^†^**
	IE	25.0		5.5	1.9	< 0.001	792.9		6.4	1.5	< 0.001
ACTH, pmol/L	O	9.7	< 0.001	14.2	5.8	< 0.001	316.9	< 0.001	15.5	5.3	< 0.001
	Combined	17.4		7.2	2.6	< 0.001	554.9		10.4	2.8	< 0.001
	IE	1033.5		5.7	0.3	< 0.001	873.5		4.5	4.1	0.4
Cortisol, nmol/L	O	411.6	< 0.001	22.4	4.9	< 0.001	625.8	0.060	5.2	4.8	0.8
	Combined	722.5		9.0	0.6	< 0.001	749.7		4.5	4.2	0.7
	IE	22.8		4.3	6.9	0.016	116.5		4.5	6.3	0.14
PTH, pmol/L	O	29.9	0.004	2.8	4.8	0.022	125	0.660	3.8	4.9	0.2
	Combined	26.3		3.3	5.7	0.014	120.7		4.0	5.1	0.2
	IE	119.3		4.5	3.4	0.2	328.4		5.1	3.8	0.15
Prolactin, μg/L	O	78.2	< 0.001	7.0	5.1	0.005	202.8	0.003	6.5	5.4	0.5
	Combined	98.8		5.4	4.2	0.2	265.6		6.1	4.4	0.074
Insulin, pmol/L	O	109.6	NA	NA	4.7	NA	135.3	NA	NA	4.6	NA
Testosterone, nmol/L	O	5.6	NA	NA	5.0	NA	188.9	NA	NA	4.9	NA
Thyroglobulin, μg/L	O	20.8	NA	NA	5.1	NA	207.9	NA	NA	4.7	NA
*Statistical significance for delta check limits was determined using the quantile regression (*e.g.* for absDD of ACTH, the DCL of IE which is 25.0 and DCL of O which is 9.7 were tested with quantile regression). ^†^Statistical significance between groups was determined using Chi-squared analysis (e.g. for absDD of ACTH, the proportion of 7.2% and 2.6% were tested with Chi-square analysis). P < 0.05 was considered statistically significant. DCL - delta check limit. absDD - absolute delta difference. absDPC - absolute delta percent change. ACTH - adrenocorticotropic hormone. PTH - parathyroid hormone. IE - inpatients/emergency. O - outpatients. NA - not applicable.											

For insulin, testosterone, and thyroglobulin, the IE group was excluded from analysis due to a sample size of fewer than 1000, therefore analysis was conducted solely on the O group. The proportion outside the DCLs ranged from 4.6% to 5.1%, closely aligning with the expected proportion outside the determined DCLs (5%).

When DCL values were compared between group IE and O for ACTH, cortisol, PTH, and prolactin, quantile regression indicated a P < 0.05 in all comparisons of absDD. However, a P < 0.05 was only noted for ACTH and prolactin for absDPC. Validation using each group’s V set showed that approximately 5% (4.3 to 6.4) of results fell outside the determined DCLs for both absDD and absDPC, aligning with expectations as the DCLs were set at a 5% cutoff.

In the IE group for PTH, the lowest proportions outside the DCL were observed, with 4.3% for absDD and 4.5% for absDPC. For ACTH, the highest proportions outside the DCL among all hormones were seen, with 5.8% for absDD in the O group and 6.4% for absDPC in the IE group (Table 2).

For ACTH, cross-validation, applying IE-based DCLs to group O and O-based DCLs to group IE, for both absDD and absDPC largely deviated from the expected proportion outside the DCLs (Table 2). For cortisol, while cross-validation with absDD deviated from the expected proportion outside the DCLs (22.4% in group IE and 0.3% in group O), cross-validation using absDPC was close to 5%. For PTH and prolactin, cross-validation results showed a slight deviation from the expected proportion outside the DCLs for both absDD and absDPC (Table 2).

For ACTH, using a combined approach in common with absDD and absDPC for DCL deviated significantly from the expected proportion outside the DCLs. By contrast, cortisol’s proportion outside the DCLs with absDD alone exceeded 5%, whereas it approached 5% when absDPC was used. For both PTH and prolactin, applying combined DCLs resulted in a proportion outside the DCLs close to 5%. The Chi-squared analysis confirmed these results, correlating with the extent of deviation from the 5% target for each test group. ACTH showed a large deviation from the 5% mark using both absDD and absDPC, resulting in a highly significant Chi-squared P-value of < 0.001 for each test group. For cortisol, the P-value was < 0.001 with absDD, indicating significant deviation; absDPC achieved a P-value of 0.700, indicating no significant deviation. In the case of PTH, the P-value for absDD was 0.014, indicating a slight deviation close to 0.05. All other tests with P-values > 0.05 indicated no significant deviation, as detailed in Table 2.

The 2.5th and 97.5th percentiles for both DD and DPC, stratified by male and female, have been calculated and included in supplementary Table 1. The difference of DCL between male and female was most prominent in prolactin using absDD.

Table 3 shows the CV_I_, the average CV_A_ calculated from QC materials at each hospital, RCV_95%_, and RCV_99%_ values for each hormone test except ACTH. Thyroglobulin had the lowest CV_I_ (10.9%) but the highest CV_A_ (2.93%), whereas prolactin had the highest CV_I_ (45.0%) but the lowest CV_A_ (1.95%). For all six hormone tests, RCV values for increases were consistently higher than those for decreases. The RCV_95%_ increase values were all lower than the combined absDPC limit, while the RCV_99%_ increase values were higher than the absDPC limit for prolactin and insulin.

**Table 3:** Reference change values for 95% and 99% probability of each hormone test except adrenocorticotropic hormone

**Parameter**	CV_I_*, % (lower CI limit, higher CI limit)	**CV_A_^†^, % (95% CI)**	**RCV_95%_^‡^, % (95% CI)**		**RCV_99%_^§^, % (95% CI)**	
			**Decrease**	**Increase**	**Decrease**	**Increase**
Cortisol	16.1 (15.5, 26.6)	2.12 (2.02 to 2.22)	- 36.1 (- 35.0 to - 51.7)	56.5 (53.9 to 106.9)	- 44.4 (- 43.2 to - 61.5)	79.9 (76.0 to 159.5)
PTH	14.7 (11.3, 25.9)	2.19 (2.07 to2.31)	- 33.7 (- 27.2 to - 50.8)	50.7 (37.4 to 103.2)	- 41.6 (- 34.1 to - 60.5)	71.3 (51.7 to 153.3)
Prolactin	45.0 (39.2, 58)	1.95 (1.85 to 2.04)	- 69.6 (- 65.0 to - 77.5)	229.0 (185.6 to 345.3)	- 79.0 (- 74.7 to - 85.9)	376.5 (295.9 to 608.8)
Insulin	25.4 (21.1, 37.1)	2.54 (2.41 to 2.67)	- 50.2 (- 44.2 to - 63.1)	100.9 (79.1 to 171.3)	- 59.9 (- 53.4 to - 73.0)	149.6 (114.7 to 270.0)
Testosterone	14.5 (10.9, 16.3)	2.41 (2.32 to 2.50)	- 33.3 (- 26.5 to - 36.6)	49.8 (36.1 to 57.8)	- 41.2 (- 33.3 to - 45.0)	69.9 (49.9 to 81.8)
Thyroglobulin	10.9 (10.3, 16.2)	2.93 (2.76 to 3.09)	- 26.7 (- 36.5 to - 25.6)	36.5 (34.5 to 57.4)	- 33.5 (- 32.2 to - 44.8)	50.4 (47.4 to 81.2)
*Median CV_I_ estimate (lower CI limit - higher CI limit) last updated on the European Federation of Clinical Chemistry and Laboratory Medicine Biological Variation Database on Sep 4th 2024. ^†^CV_A_ used in the calculations was determined as the average of CV_A_s obtained from quality control materials at each hospital. ^‡^ RCV with 95% probability calculated using 1.96 as two-sided approaches Z-score. ^§^ RCV with 99% probability calculated using 2.57 as two-sided approaches Z-score. PTH - parathyroid hormone. CV_I_ - within-subject biological variation. CV_A_ - analytical imprecision. CI - confidence interval. RCV - reference change value.						

## Discussion

This study is the first to establish DCLs for frequently requested hormones, aiming for a 5% proportion outside the defined limits based on patient result distributions. Significant differences in DCLs were observed between the IE and O groups for ACTH, cortisol, PTH, and prolactin, with ACTH and cortisol displaying DCLs that could not be applied interchangeably across groups. Combined DCLs, calculated from the average of IE and O group values, showed consistent proportions outside the DCL for cortisol, PTH, and prolactin when using absDPC, supporting the feasibility of combined DCLs for these hormones.

Delta checks are a method used to compare current and previous test results to identify significant discrepancies, which can help detect analytical or preanalytical errors. While delta checks are commonly used for various biochemical tests, their application to hormone assays is less frequently documented in the literature, possibly due to the inherent diurnal and physiologic variation of hormones (*22*). For example, ACTH and cortisol exhibit significant fluctuations throughout the day, insulin has a circadian component, prolactin secretion is regulated by the circadian clock, and testosterone shows diurnal patterns (*23*-*28*). Despite these challenges, implementing autoverification rules, including delta checks, can reduce the turnaround time and improve efficiency in handling problematic test results. Therefore, research on the DCLs for hormones is necessary, and our results will provide foundational data to help establish DCLs for hormone tests in clinical laboratories (*29*).

Inpatients and patients in the emergency department often experience rapid changes in laboratory tests due to acute conditions, while outpatients typically have more stable conditions, leading to narrower DCLs for outpatients (*5*). However, contrary to other tests, PTH exhibited a larger DCL in outpatients than in the IE group. This can be attributed to the higher proportion of nephrology patients among outpatients (39.2% of outpatients *vs.* 7.2% of inpatients), many of whom are likely receiving dialysis (*30*). Patients on dialysis experience significant PTH variability due to intermittent dialysis sessions and varying compliance with phosphate binders and dietary restrictions (*31*).

This study observed that absDPC demonstrated tolerance in adjusting the DCL between groups IE and O for cortisol, PTH, and prolactin. When selecting a DCL using either absDD or absDPC, several factors must be considered. The absDPC tends to be higher when the average test result is lower, which helps align DCLs across groups with different baseline concentrations. Given its greater tolerance for cross-validation and combined DCL validation, absDPC might be preferable for setting DCLs in a simplified approach.

Testosterone and prolactin typically show significant differences between men and women, as reflected in the DCLs presented in supplementary Table 1. While setting different DCLs for each sex might be less practical in some cases, implementing sex-specific DCLs for testosterone and prolactin could enhance the accuracy of detecting abnormal variations for these hormones.

Previous studies on tumor markers suggest that, even when results are outside the determined DCLs, further investigation may be unnecessary if both the current and previous results are within the reference intervals (RIs) (*5*). Accordingly, designing algorithms that exclude delta checks when values are within the RIs would be beneficial. Although this study did not directly evaluate whether both current and previous results were within the RIs, it is reasonable to propose that, in practical laboratory settings, applying DCLs may be redundant when patient results are within RIs.

Thyroglobulin has a low CV_I_ compared with other hormones and a high between-subject biological variation (CV_G_); with a CV_G_/CV_I_ ratio of 7.3, making it a suitable candidate for delta checks (*19*). Additionally, thyroglobulin is a precursor to thyroid hormones and a crucial tumor marker for differentiated thyroid carcinoma (*3*, *32*). In this study, we provide absDD and absDPC as DCLs for thyroglobulin in outpatients. Previous study on tumor markers such as alpha-fetoprotein, carbohydrate antigen (CA) 19-9, CA 125, carcinoembryonic antigen, and prostate-specific antigen has shown that DPC is an effective delta check method for tumor markers (*5*). Considering thyroglobulin’s role as a tumor marker, absDPC may serve as an effective and practical DCL in the clinical setting.

The RCV is known as a useful delta check method for monitoring clinically significant changes (*12*). Previous studies have also highlighted that RCV typically provides a narrower range than DPC, making it less suitable for detecting sample or analysis-related errors (*5*). In this study, the combined absDPC limits for all hormones were found to be higher than the RCV_95%_ values. For prolactin and insulin, the RCV_99%_ values exceeded the absDPC limits, suggesting a broader range of clinical variation for these hormones.

This study has several limitations. First, we only included results from Roche analyzers, which may restrict the applicability of the findings to laboratories using different analyzers. Second, the lack of detailed information on the clinical conditions of the patients limited our ability to consider underlying conditions or specific clinical situations that could impact hormone concentrations and delta check. Additionally, we could not consider sample collection times due to the limited data, thereby overlooking the influence of circadian rhythms and other temporal factors on hormone concentrations. While each laboratory participated in the EQA programs of the KEQAS, a direct interlaboratory comparison was not conducted. Lastly, this study does not include an external validation of the proposed DCLs.

Traditional delta check methods are simple and crude, and thus lack high sensitivity and specificity. Recent reports indicate that machine learning can offer better performance in this area (*6*). Future research should explore the application of machine learning-based delta checks incorporating multiple parameters for hormone assays. Despite these limitations, this study is significant in its attempt to determine DCLs for various hormones, a previously unexplored area.

In conclusion, we established DCLs for frequently requested hormones using real-world data. We suggest using absDPC as a combined DCL for cortisol, PTH, and prolactin, whereas different DCLs are based on clinical settings for ACTH. These results will provide foundational data to help establish DCLs for hormone tests in clinical laboratories.

## Data Availability

The data generated and analyzed in the presented study are available from the corresponding authors on request.
